# Implementation of a methodological protocol for the national survey on tuberculosis catastrophic costs in Brazil

**DOI:** 10.1590/0037-8682-0493-2022

**Published:** 2023-02-20

**Authors:** Ethel Leonor Noia Maciel, Leticya dos Santos Almeida Negri, Leticia Molino Guidoni, Geisa Carlesso Fregona, Rafaela Borge Loureiro, Isadora Bianchi Daré, Thiago Nascimento do Prado, Mauro Niskier Sanchez, Fredi Alexander Diaz-Quijano, Maiko Tonini, Eliana Zandonade, Inés Garcia Baena, Julia Ershova

**Affiliations:** 1 Universidade Federal do Espírito Santo, Laboratório de Epidemiologia, Vitória, ES, Brasil.; 2 Hospital Universitário Cassiano Antônio Moraes, Programa de Tuberculose, Vitória, ES, Brasil.; 3 Universidade de Brasília, Faculdade de Ciências da Saúde, Departamento de Saúde Coletiva, Brasília, DF, Brasil.; 4 Universidade de São Paulo, Faculdade de Saúde Pública, Departamento de Epidemiologia, São Paulo, SP, Brasil.; 5 Ministério da Saúde, Programa Nacional de Tuberculose, Brasília, DF, Brasil.; 6 Universidade Federal do Espírito Santo, Ciências da Saúde, Departamento de Estatística, Vitória, ES, Brasil.; 7 World Health Organization, Global TB Programme, Geneva, Switzerland.; 8 U. S. Centers for Disease Control and Prevention, Atlanta, USA.

## INTRODUCTION

The World Health Organization (WHO) has set goals to end the tuberculosis (TB) pandemic by 2035, that is, to reduce the incidence to less than 10 cases per 100,000 inhabitants and the mortality rate by 95%. Although TB is a disease affecting people of all socioeconomic classes, it is mainly found in marginalized and vulnerable populations^1^. In 2019, before the onset of the coronavirus disease 2019 pandemic (*COVID-19)*, 10 million people were diagnosed with TB worldwide, 7.1 million were reported to public health services, and 1.4 million died of the disease, including 20,800 people with human immunodeficiency virus (HIV)^2^ ^,^ ^3^.

Brazil is one of 30 countries with the highest burden of TB; the incidence of TB in 2020 was 36.83 per 100,000 population, ranging from 24.85 in Santa Catarina to 76.97 in Amazonas. Meanwhile, the mortality data revealed 4,900 deaths due to TB in Brazil in 2019. The TB mortality rate is alarming in Pernambuco (4.11 per 100,000 inhabitants), compared with that in Paraná (1.11 per 100,000 inhabitants)^4^ ^-^ ^6^.

Meeting the targets that were established by the WHO and agreed upon by the Ministries of Health to reduce TB incidence and to improve its indicators is an ambitious challenge. The End TB Strategy has set three high-level indicators to facilitate progress towards achieving global TB targets^1^, one of which is to ensure that neither people with TB nor their households face “catastrophic total costs” due to TB. Costs are defined as catastrophic when patients spend 20% or more of their annual household income on TB diagnosis and care.

To measure and monitor the catastrophic costs related to TB, the WHO recommends that countries with a high burden of TB regularly conduct nationwide population-based patient cost surveys. To standardize the methodology and measure the proportion of TB-affected households experiencing catastrophic costs nationwide, the WHO developed a generic protocol and survey instrument, which was subsequently used by 24 countries as of August 2021^7^. The proportion of TB-affected households experiencing catastrophic total costs ranged from 13% (95% confidence interval [CI]: 10-17%) in El Salvador to 92% (95% CI: 86-97%) in the Solomon Islands.

From 2019 to 2021, we conducted the first nationally based TB patient cost survey in Brazil using the WHO methodology to assess the catastrophic costs faced by households owing to TB and to build a catastrophic cost indicator. The WHO generic protocol and data collection tools were adapted for Brazil using the Critério de Classificação Econômica Brasil and the Pesquisa de Orçamentos Familiares (POF) Simplified Model IV (translated as *Brazil Economic Classification Criteria* and *Household Budget Survey,* respectively)^8^ ^-^ ^9^.

This manuscript describes the Brazil TB patient cost survey protocol, including the study design, population, and sample size calculation, as well as our experience implementing the survey, including patient enrollment and data analysis. 

## METHODS

### Study design and population

We aimed to conducted a nationally representative health facility-based survey to assess the economic burden of patients with TB in Brazil during TB diagnosis and treatment. The data were collected retrospectively based on patients' recall (self-report) at the current treatment phase; the calculated cost was extrapolated within the treatment period according to the standard WHO methodology^1^.

The survey population included patients with TB of all ages with any type of TB (e.g., pulmonary or extrapulmonary, new or retreated, and drug-susceptible (DS) or drug-resistant (DR) TB) who were registered for TB treatment under Brazil's National TB Program (NTP). Patients who registered for TB treatment in the facilities linked to the NTP and who visited a sampled facility for treatment during the study period were eligible to participate in the survey, according to the sampling strategy. Legal guardians represented children under 18 years of age.

Incarcerated persons, unaccompanied minors, and those being treated for less than 14 days in the intensive or continuation phase were not enrolled in the study.

The survey objectives were as follows:

1. Documentation of the magnitude and main drivers of different types of costs incurred by people with TB and their households.
2. Determination of the proportion of people with TB (and their households) registered in the NTP who are experiencing catastrophic costs (costs exceeding 20% of the annual household income) owing to TB diagnosis and treatment.
3. Initiation of future research to develop or scale up policies and interventions so as to reduce the costs associated with TB.

### Sample size calculation

We used a cluster-based approach and a probability proportional to size sampling strategy to calculate the sample size and select the clusters. Each cluster was represented by a municipality. The sample size calculation was based on the number of TB notifications in 2017 (N = 88,040), design effect (DEFF) = 2, and an absolute precision of 5%. On the basis of preliminary results from TB patient cost studies in Brazil and other countries, we anticipated that approximately 40% of people with TB would incur catastrophic costs (more than 20% of the annual household income) owing to TB diagnosis and treatment in Brazil^10^. These costs could be higher for poor people and those with DR-TB, especially multidrug-resistant TB (MDR-TB) or extensively drug-resistant TB (XDR-TB). 

The sample computation was based on the cluster sampling formula below, as recommended by the WHO handbook for TB patient cost surveys^1^.

$$n=\text{DEFF}x\frac{1.96^{2}N\left(1-\pi_{g}\right)\pi_{g}}{d^{2}(N-1)+1.96^{2}{\left(1-\pi_{g}\right)\pi_{g}}_{\;}}$$

where,

**Table:** 

*n*	Number of people included in the patient survey
$\pi_{g}$	Anticipated catastrophic cost prevalence (“prior guess” of the true proportion of families experiencing catastrophic total costs owing to TB)
*d*	Absolute precision (expressed as a proportion)
*N*	TB population size
DEFF	Design effect

The survey sample size was 760 patients with TB who were recruited from 46 clusters.

### Cluster’s stratification

We applied the stratification technique when selecting clusters/municipalities. The municipalities were divided into two groups: group 1 was composed of municipalities with ≥ 35 reported cases in 2017 and group 2 was composed of < 35 cases. Thus, the 760 cases were classified as follows: 720 (95%) cases in group 1 (36 municipalities, 20 cases each) and 40 (5%) cases in group 2, proportional to the number of patients treated in each group. Furthermore, because the characteristics of people treated in primary healthcare (PHC) and special care (SC) facilities are different, both facility types should be properly represented in the study. As such, the survey strata represented the capacity (≥ 35 or < 35 reported cases in 2017) and type of healthcare facility (PHC or SC). Under- and over-enrolment were adjusted at the time of analysis.

### Survey training and pilot

Interviewer training is key to successfully implementing the survey. Therefore, the survey team conducted a 3-day training for all the staff involved in the survey. Interviewers were selected from among the students and staff of the Federal University of Espirito Santo (UFES). Knowledge of local Brazilian languages was preferable but not required for the selection. The training was conducted by investigators from the UFES and NTP, as well as consultants from the WHO, Pan American Health Organization, and the Centers for Disease Control and Prevention. The training covered survey methodology, protocol details, and good clinical practice. The objectives of the interviewers’ training were as follows:

- To be informed about the nature of TB and infection control procedures for personal protection during the interviews
- To gain awareness of ethical issues in performing such interviews
- To be familiar with the electronic data collection tool
- To learn interviewing techniques (such as adequate probing)
- To be able to select the eligible study participants
- To be familiar with the survey questionnaire (data collection tool)
- To understand the indicators used in the questionnaire, particularly the different phases and types of TB treatment and associated costs, so as to avoid double counting
- To understand TB drugs and other drugs or vitamins that can be prescribed to or purchased by the patient
- To share feedback on any uncertainties or concerns with the questionnaire, data, or collection procedures with the survey coordinator

During the training, interviewers applied the questionnaire with each other and in simulated facilities to ensure that they understood the questions and responses.

After the training, survey interviewers were able to do the following:

- Introduce themselves and the survey to the potential participant
- Justify the inclusion criteria for the survey to the potential participant
- Convey to the potential participant the informed consent process
- Check the participant’s medical records
- Put the participant at ease and ensure a comfortable environment in which to ask questions
- Be familiar with the questionnaire, so that questions are asked conversationally rather than stiffly
- Ask questions in the order in which they are written in the questionnaire, using the same wording (in Brazilian Portuguese). Certain questions may require further explanation or prompting from the participant, especially regarding time and types of costs.
- Facilitate interviewee recall using local methods of time structuring. Depending on how far the participants have progressed with treatment, they might find it difficult to recall item cost. For example, if the patient has sold livestock to pay for travel and treatment, the interviewer may explore the approximate price based on the local context if the respondent cannot recall the price.
- Understand and be able to explain indicator definitions, types of costs, what is meant by the cost of food, travel, and accommodation, what is included and excluded, and how they can help patients recall items by prompting. This will help ensure consistency in interviews and prompting by interviewers.
- Avoid influencing the answers to questions by using friendly but neutral body language and not educating the participant
- Ensure that all questions are answered. They must add “zero” in a column if no money was spent on an item (did not pay any money) and select “no answer” if the question was not answered (patient did not remember).
- Keep control of the interview (avoid off-track conversations and long silences)
- Be sensitized on the different phases (i.e., intensive and continuation) and types of TB treatment (e.g., hospitalization and different forms of directly observed therapy [DOT]) and associated costs (e.g., sputum conversion test, follow-up test, and medicine collection) to avoid double-counting costs. They must be informed about the nature of TB, what their participation means for their health, and how they can protect themselves.
- Gain knowledge on infection control procedures
- Distinguish the type of interview (i.e., person with TB or legal guardian of the person with TB) to ensure that the questions are related to the person with TB

The training was conducted by experienced NTP, WHO, and CDC staff, as well as academic staff from the University of Espirito Santo. Some trainers from these agencies have been involved in previous TB patient cost surveys. After the training, a pilot test was conducted to examine interviewers’ knowledge of the entire process of patient enrollment, interviewing, and data entry. The pilot test was conducted in two steps. First, we tested the functionality of the questionnaire by asking the local TB program staff to collect “dummy” data. This step instilled confidence that the questionnaire functioned properly, and that the TB program managers and staff were familiar with and agreed with the questionnaire’s content.

Second, we conducted a pilot survey in the field before starting the survey in the two clusters that were not included in the list of selected facilities. Pilot testing provided an opportunity to identify problems with the survey tools, the time required for the interviews, and the necessary budget. The wording, sequence, and structure of the questionnaire were adjusted according to the pilot test findings. 

### Patient enrollment

Consequent patients with TB who attended the selected facility for follow-up visits on the day of the survey team’s visit were enrolled in the study according to eligibility criteria until the cluster size had been reached^1^. Patients and legal guardians were interviewed only once; no re-interview was conducted. Some patients were interviewed in the intensive treatment phase, and others in the continuation treatment phase. We anticipated recruiting half (50%) of the sample in the intensive phase and half (50%) in the continuation phase in each cluster to achieve a good balance between the two groups and to ensure a sufficient number of people reporting pre-treatment cost data, which were only collected from people in the intensive phase. 

In agreement with the health facility, a planned interview was scheduled after identifying people eligible for the survey to avoid missing opportunities for interviewing patients who were close to completing treatment when surveying started and to avoid interviewer downtime. 

Each potential survey participant was adequately informed about the study using Brazilian Portuguese or other ethnic languages and was asked to sign an informed consent form before enrollment. The information provided to the potential survey participants included the following:

- The purpose, methods, and procedures of the survey
- Possible discomforts involved
- The right to abstain from participation in the survey or to withdraw consent at any time without reprisal
- The sources of funding of the survey and institutional affiliation of the researchers
- Description of how anonymity or confidentiality would be protected
- The amount of compensation for participating in the survey
- The extent to which the results will be made available to the participant or the community

### Data collection strategy and tool

The interview questionnaire included questions about the sociodemographic, clinical, and economic status of the patients and their households, including individual and family income before and during treatment, household goods, family food security, direct medical payment (e.g., consultation fee, laboratory tests, and medications), non-medical expenses (food, accommodation, and transportation), and indirect costs (loss of income or cost of time as a result of TB treatment) (Table 1). Questions on coping strategies, social consequences, and household consumption were also included. The questionnaire was adopted from the WHO generic questionnaire for TB patient cost surveys and translated into Brazilian Portuguese^11^.

**TABLE 1:** Information collected during the interview.

Collected information
Clinical parameters
Demographic variables, employment, and household composition
Socioeconomic position
Healthcare utilization (in all types of institutions for pre-treatment and NTP network facilities and non-penitentiary facilities during treatment)
Time spent and income lost while seeking and receiving care
Direct medical costs, direct non-medical costs, and indirect costs
Household and individual income
Coping mechanisms (loans taken, assets sold, etc.)
Access to social protection
Social consequences and perceived impacts of costs

**FIGURE 1:**
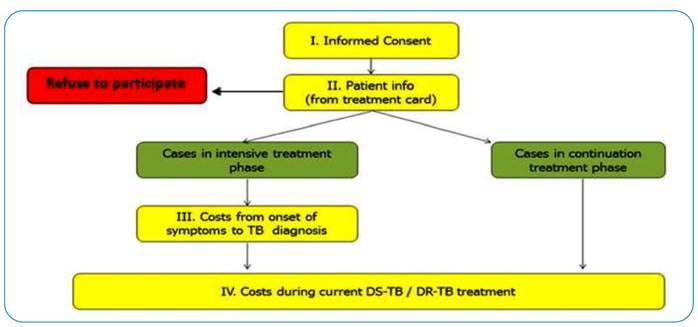
Flow of questionnaire components for the tuberculosis cost survey in Brazil (TB, tuberculosis).

The data collection tool included four components, as outlined in Figure 1. 

Information from the TB treatment record (Part II) and information about costs related to the current TB treatment (Part IV) were collected from all eligible patients after they signed the informed consent form (Part I). 

Information about costs and income loss related to health-seeking and diagnostic procedures from the onset of TB symptoms until diagnosis and registration with TB within the NTP network (Part III) was collected only from those interviewed in the intensive phase. This strategy recognizes the challenge of remembering events and costs incurred many months before the interview. In addition, people in the intensive phase of TB treatment also reported on the costs during the “current” intensive phase (Part IV).

Conversely, for people interviewed in the continuation phase, the information collected was limited to the costs and time loss experienced during the continuation phase, except for one question related to reporting household income at the time of diagnosis. 

Information collected in Part III for people interviewed in the intensive phase was used to impute data and estimate the costs for those interviewed in the continuation phase. Similarly, information about costs in the continuation phase, collected from people interviewed in this phase, was used to project the continuation phase costs for those interviewed in the intensive phase. 

To estimate the costs in the remainder of the patient’s current treatment phase, we extrapolated the patient’s costs in the treatment phase to date. For example, if only one-third of the treatment phase had been completed, the costs to date in the treatment phase were multiplied by three. This extrapolation was performed using information about the planned phase duration and phase days completed. To estimate costs from the other phases of treatment, we used the median reported costs and the number of hours from other patients sampled in that treatment phase. In this calculation, the costs of patients with DS-TB and MDR-TB were considered separately. 

### Household income measurement to estimate a household’s capacity to pay for health

The household capacity to pay for health can be estimated in several ways. For example, income based on self-reported household consumption (Measure 1), estimated income based on reported asset ownership (Measure 2), and self-reported income (Measure 3)^1^. Currently, there is no consensus on the best method for estimating income in the context of a survey at a health facility. Recent literature demonstrates that the proportion of patients encountering catastrophic costs varies according to the method used^12^.

While reported income is the most straightforward measure of a household’s ability to pay, in countries with a large informal work sector (such as Brazil), monthly self-reported household income is subject to limitations. For example, income may fluctuate significantly if patients rely on intermittent, casual, or seasonal work. In addition, self-reported income is open to recall and reporting bias (especially when respondents do not receive a regular pay slip), potentially leading to over or underestimation by respondents. In settings where employment is mainly outside the formal sector, and income is difficult to measure reliably, consumption as a measure of income is believed to be a more valid measure of household income^12^. Therefore, in Brazil, the measurement of consumption could be a more accurate measure of income than self-reported income^13^. According to previous studies on the economic consequences of TB, a disproportionate number of participants were unable to provide self-reported income data, and self-reported mean annual income was significantly lower than permanent income estimated using other approaches, such as consumption or household asset approaches^10^.

**FIGURE 2:**
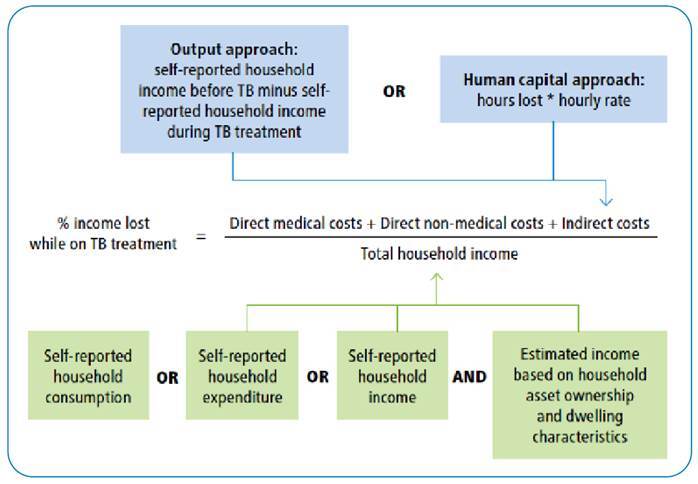
Household income measurements for estimation of the household’s capacity to pay for health (TB, tuberculosis).

An overview of the different approaches used to measure the household capacity to pay is provided in Figure 1 ^1^. Our study used a valuation of time loss (defined in the WHO TB patient cost surveys handbook as “human capital approach”) to measure indirect costs and annual household consumption measures to estimate the household’s ability to pay (Figure 2). The human capital approach involves valuing an individual’s time based on their reported income before the onset of illness (by multiplying the number of hours spent seeking and receiving care or caring for a patient by their reported hourly wage rate).

The monthly household consumption in our study represents the resources that households consume, as reported by the patient. To measure consumption in this survey, respondents were asked a series of questions about how much (if anything) their household had consumed (including home-grown products) weekly, monthly, or annually on goods and services, including food items, non-food items, durable goods (i.e., goods that last for a long time, such as motorbikes), and housing. These amounts were multiplied by 52 and 12, where relevant, and added up during the analysis to arrive at the annual household consumption.

In addition to household consumption, we estimated household income using self-reported income and household asset approaches. We acknowledge the limitations associated with these approaches; the last one is linked to the fact that assets are slow-changing and, therefore, may not capture changes in household economics accurately, particularly for the lowest quintile^13^ ^-^ ^14^.

### Estimating costs

To estimate the costs of TB treatment, we assessed the costs for patients with TB and their households in each treatment phase, including direct medical, direct non-medical, and indirect costs.

Median direct costs for DS-TB and MDR-TB survey participants per phase were calculated for the phase in which the interviewed participants were treated during the interview. We then used these results to extrapolate the direct costs for patients in another phase of treatment. Direct non-medical guardian costs were included in the calculation only if the guardian was a member of the same household as the person with TB. 

To calculate direct costs, we summarized out-of-pocket payments made by people with TB or their guardians (net of reimbursements) for medical services (i.e., medical costs) and transportation, accommodation, food, and nutritional supplements (i.e., non-medical costs) incurred in the pre-diagnosis and during-treatment phases. Pre-diagnostic direct costs included self-reported expenses on consultations, testing (e.g., X-ray), and medications, subtracting any insurance reimbursement and travel, food, and accommodation accessing healthcare services. During-treatment direct costs included self-reported monthly expenses accessing DOT services (transport and food), picking up anti-TB medicines (administrative fees, transportation, food, and accommodation), follow-up tests (administrative fees, sputum, X-rays, medications, and others), hospitalization (administrative fees, tests, medicines, sheets/clothes, food, transport, and others), and the purchase of special foods and vitamins.

Indirect costs, defined as the opportunity cost of time away from daily productive routine as a result of TB healthcare visits and hospitalization during the TB episode, can be calculated using two distinct approaches: a) self-reported household income loss net of welfare payments and b) total period of absence in hours multiplied by the hourly wage rate of the absent worker^1^. The choice of approach is highly dependent on the measure of the ability to pay for health that will be used in the denominator of the catastrophic cost indicator. For our study in Brazil, we used Measure 1 to estimate income; thus, we measured indirect costs by multiplying the reported time used while seeking and receiving care during the TB episode (in hours) by an individual hourly income^1^. Children were, by default, given an hourly wage of zero; instead, the estimated hourly income for the legal guardian(s) was used. Individual and household hourly incomes were estimated based on reported income data collected from all survey participants.

We also measured indirect costs using an alternative method and compared the results. The alternative method compared self-reported income at three time points (pre-disease, at the time of diagnosis, and during the interview).

For the total indirect cost for DOT visits per month, we used the number of visits per week multiplied by 4 (1 month) multiplied by the duration of the visit multiplied by the value of the people's work time [people work time: sickness divided by 192 (1 week = 6 working days × 8 hours = 48 hours × 4 weeks = 192 hours per month)]. For the total indirect cost of picking up medication, we used the number of visits to a health facility to take medications per month multiplied by the duration of the visit multiplied by the value of people's work hours. For the indirect cost of test visits and follow-up, we used the number of visits to the service for tests or health follow-up times and the duration of the visit multiplied by the value of the people's working time. Finally, for the indirect cost of hospitalization, we used the number of hospitalization days multiplied by the patient's income divided by 24 (1 month = 24 working days). 

Therefore, for the total indirect cost of people with TB, we used the sum of the total indirect costs due to inability to work, costs for DOT visits per month, drug withdrawal costs, testing and follow-up visit costs, and any hospitalization costs. 

Annual household consumption was used as the main approach for the End TB indicator measurement. The household consumption was calculated following the same method used by the Instituto Brasileiro de Geografia e Estatística; the relative questions were extracted from POF Simplificado, and indirect costs were measured using the time lost since the start of TB symptoms until the time of the interview. This calculation was performed by multiplying the number of hours lost during travel and waiting at the health facility and receiving care by the reported individual income per person per minute^15^.

### Estimating catastrophic costs

**FIGURE 3:**
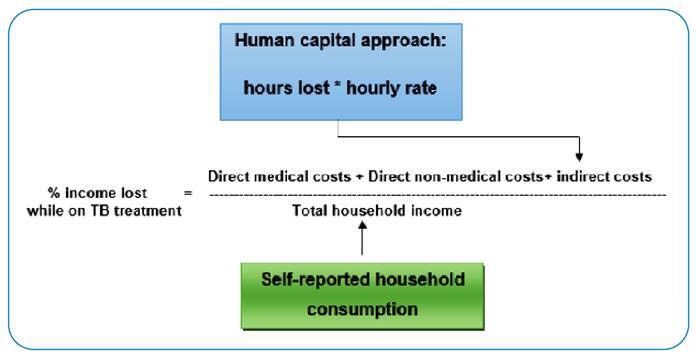
Main approach used for the Brazil survey.

For the definition of total catastrophic costs, each household received a binary value to determine whether they incurred costs due to TB diagnosis and treatment, defined by 20% of the annual consumption threshold. We also conducted a sensitivity analysis to assess the different “catastrophic” thresholds. Finally, we calculated the proportion of costs due to TB for each household according to the formula in Figure 3 and used the following WHO-recommended formula to identify households faced with catastrophic costs:

$$\frac{Episode\;direct\;cost\;+\;Episode\;indirect\;cost\;(hours\;lost\;\times \;hourly\;rate)}{Self-reported\;household\;consumption}>20\%$$

### Measurement of impoverishment

We evaluated pre-disease household poverty levels by comparing daily income (calculated from self-reported household monthly income and comparing against the international poverty threshold of US$1.90 purchasing power parity adjusted dollars (converted to Brazilian Real (BRL) in 2011, then inflated using the ratio of consumer price index values in 2011 vs. 2020) as reported in the international finance statistics database^16^.

### Coping strategies

Coping strategies in our study were defined as borrowing funds or selling assets to finance TB care expenditures. An analysis of coping strategies gave us insight into how people with TB deal with TB treatment costs. Each coping strategy was examined as a binary measure, that is, whether the household used the coping strategy and the absolute amount of coping, defined as the total amount of money borrowed/received to finance the cost incurred during TB treatment.

### Social consequences of TB

Descriptive analysis was conducted on the frequency of the social consequences during/after TB treatment, such as food insecurity, social exclusion, divorce, interrupted schooling, and job loss. The data collection tool included questions related to access to government social protection. Social protection payments were measured as the monthly value of self-reported social insurance from sickness allowance, basic and variable benefits of Bolsa Família (BPC), benefits to overcome extreme poverty from BPC, continued BPC benefits, reimbursement of private health insurance, paid sick leave, and disability benefits. 

### Statistical analysis

Data stratification was presented by the patients’ capacity for the facilities and types of services, such as Primary Care and Specialized Care. Continuous variables were analyzed using the medians, means, and 95% confidence intervals (95% CI). For categorical variables, absolute and relative frequencies were calculated. Extrapolation of costs beyond a participant’s current treatment phase was done based on the median costs incurred by other patients and their households in the alternative treatment phase at the time of the interview^1^ with a significance level of 5%. Statistical analyses and data visualizations were performed using R Studio®, version 14. All cost and income data were collected in Brazilian Real.

### Ethical considerations

TB patient cost surveys involved the administration of a questionnaire to people with TB regarding their TB diagnosis, treatment, and associated costs. There are a few risks associated with this kind of study, as it only involves administering a questionnaire using trained interviewers. Potential risks included distress in participants financially or socially affected by TB because of recalling related events. Thus, if the participant appeared distressed, the interviewer temporarily stopped the interview. The interviewer had the option of referring the person to a nurse or doctor if further psychological support was needed. Additionally, there was a small risk that someone might be identified based on the responses provided to the interviewer. To mitigate the risk of someone being identified based on their responses, all information was kept in a password-protected database accessible only by the study team. In addition, presentations or publications related to the study contained only summarized and de-identified data. Although people with TB were interviewed two weeks after TB treatment initiation, there was a risk of TB infection from participant to interviewer, as patients with DR TB may not be on appropriate treatment due to delays in laboratory results. Thus, as interviewers were aware of any risk and knowledgeable about infection control measures, patients with MDR-TB were interviewed outside or in a well-ventilated room with N95 respirators. 

The catastrophic cost survey in Brazil underwent three ethical evaluations at the Conselho Nacional de Ética em Pesquisa - CONEP (National Council for Research Ethics) with No. 4,452,641, by the World Health Organization (No. PAHOERC-2019-0026), and the Center for Global Health of the U.S. Centers for Disease Control and Prevention (CGH HSR Tracking #:2019-167).

### Protocol limitations

The protocol has some limitations inherent in the WHO methodology for implementing TB patient cost surveys. A major challenge in estimating patient costs is recall bias; patients do not accurately remember the amount of time or money they spent on TB diagnosis and treatment. This predominantly affects the cost estimates for the pre-treatment period. The WHO-suggested approach to only interview persons in the intensive phase about diagnostics costs is intended to minimize this type of bias. The definition of catastrophic cost depends on the measurement of another variable, household income, which can introduce several sources of error, including under-reporting of undeclared earning resources. However, the impact of TB costs may differ from one socioeconomic setting to another where families live. The survey results could only be extrapolated to patients with TB who received care in NTP facilities. However, the majority of patients with TB in Brazil are registered for treatment in NTP-linked facilities. Finally, we did not interview the relatives of the patients who were “lost to follow-up,” and some of these patients might have disrupted treatment due to financial difficulties. Therefore, the proportion of patients with catastrophic costs may have been underestimated. We also did not include households with more than one patient with TB. We did not interview the relatives of patients who had died due to TB because of our limited resources and the complexities of conducting such a survey in Brazil. We acknowledge that this is an important area for future research and is also recognized by the WHO.

Despite these limitations, the TB patient cost survey protocol in Brazil established a methodology for calculating and monitoring the End TB Strategy Catastrophic Cost Indicator. This allowed the cost surveys, which provide important information for programming and policy development, to be repeated regularly. 

### Ethics approval and consent to participate

The survey of catastrophic costs in Brazil was approved by the institutional Ethics Committee, together with the consent to participate in the research, in which all patients signed an informed consent form. 

The catastrophic cost survey in Brazil underwent three ethical evaluations at the Conselho Nacional de Ética em Pesquisa - CONEP (National Council for Research Ethics) with No. 4,452,641, by the World Health Organization (No. PAHOERC-2019-0026), and the Center for Global Health of the U.S. Centers for Disease Control and Prevention (CGH HSR Tracking #:2019-167).

## RESULTS AND DISCUSSION

We developed a methodological protocol and executed the first national population-based survey to measure the costs faced by patients with TB and their households in Brazil. All previous studies that aimed to evaluate the costs of TB diagnosis and treatment and develop strategies for preventing catastrophic costs for families in Brazil were conducted locally using different methodologies^10^ ^,^ ^13^ ^,^ ^17^ ^-^ ^19^. Our protocol closely follows the standard WHO methodology and provides an opportunity to repeat cost surveys regularly to monitor progress toward the End TB Strategy target of eliminating catastrophic total costs owing to TB in Brazil. Using a standard methodology for assessment and monitoring, the catastrophic cost indicator improves data quality, ease of replication, and internal and external validity, allowing for the production of reliable data on the magnitude and drivers of TB-related costs for patients and their households. This information is essential for developing appropriate policies and interventions to reduce the cost related to TB care, which will contribute to improving access and adherence to treatments and protecting against economic hardships. The survey findings will be used to monitor financial access barriers and inform related health and social policy changes to improve TB prevention and care^7^. The standard methodology will also allow comparisons of Brazil’s survey results with those of other countries, provide a basis for further analysis of the economic impact of TB, and inform policymakers in Brazil about the progress of implementing the social protection program at a national level.
